# Neural Correlates of Self-Construal Priming in the Ultimatum Game

**DOI:** 10.3389/fnins.2019.00994

**Published:** 2019-09-24

**Authors:** Nic Flinkenflogel, Tuong-Van Vu, Marlieke T. R. van Kesteren, Lydia Krabbendam

**Affiliations:** ^1^Department of Clinical, Neuro- and Developmental Psychology, Institute for Brain and Behavior Amsterdam, Vrije Universiteit Amsterdam, Amsterdam, Netherlands; ^2^Department of Education Sciences, Faculty of Behavioral and Movement Sciences, Institute for Brain and Behavior, Vrije Universiteit Amsterdam, Amsterdam, Netherlands

**Keywords:** fMRI, self-construal, priming, decision-making, ultimatum game

## Abstract

Research from cultural and social psychology has identified a central role of self-construal, or the way one views themselves in relation to others, in social cognition. Accordingly, it is plausible that self-construal plays an instrumental role in important aspects of decision-making relating to fairness considerations. Prior research has shown that priming methodology is a useful tool to experimentally isolate the effect of self-construal on social decision-making processes. In the current study we investigated the neural effects of self-construal priming on fairness considerations, using an Ultimatum Game setup (*N* = 97). Based on previous findings, we predicted an interaction between the self-construal prime and gender on Ultimatum Game behavior; males primed with interdependence would reject the offer relatively more compared to independence, and vice versa for females. As previous neuro-imaging research has established an instrumental role of the anterior insula (AI) and ventromedial prefrontal cortex (vmPFC) in the rejection of unfair offers, we expected higher rejection rates to be mirrored by increased activity in these regions. However, the analyses did not confirm these predictions. As further inspection of the data revealed a habituation effect, we performed a follow-up analysis on the first block (*N* = 59). This subsequent analysis revealed that priming interdependence resulted in reduced AI activity compared to priming independence, although no behavioral differences were observed. The difference was theorized to result from motivations as conflict avoidance and harmony maintenance, commonly associated with interdependence. Furthermore, the analysis revealed greater vmPFC activity for females compared to males for rejected offers, although this effect was not robust when controlled for trait self-construal. These follow-up analyses suggest that self-construal priming influences insula activity, as well as implicating an underlying role of trait self-construal in observed gender differences in vmPFC activity relating to fairness considerations.

## Introduction

The role of fairness in social and economic decision-making has been a central topic in psychological research over the past few decades (Kahneman et al., 1986). Fairness considerations are derived from social norms; a predefined set of expectations that govern how we are supposed to behave, and what can be expected from others (Buckholtz and Marois, 2012). Accordingly, social norms can be viewed as a cornerstone of human society, with an instrumental role of punishment in maintaining human’s uniquely cooperative nature (Fehr and Fischbacher, 2004). However, while the desire for fairness (e.g., justice, equality and equity) is a relatively universal construct (Decety and Yoder, 2017), there is simultaneously large variation at the individual and cultural level in what is considered fair (Henrich et al., 2001; Oosterbeek et al., 2004). Accumulating research from social and cultural psychology has suggested that individual differences in self-construal, or the way one defines him- or herself in relation to others, may partly explain this variation; e.g., individuals that construct an independent self-make choices that primarily promote their own well-being, while those that construct an interdependent self-incorporate the goals and motivations of relevant others in their decision-making and behavior (Gelfand et al., 2002; Gollwitzer and Bücklein, 2007; Cross et al., 2011). However, several studies have suggested that the effects of self-construal may be moderated by gender (Maddux and Brewer, 2005; Van Vugt et al., 2007; Flinkenflogel et al., 2017). For example, by using self-construal priming methodology, males were found to display opposing behavior compared to females relative to the self-construal primes (Flinkenflogel et al., 2017). The aim of this study was to further examine the relationship between self-construal and gender relating to fairness, while investigating the underlying neural correlates using functional neuroimaging.

Self-construal was first described as a manifestation of the cultural syndromes of individualism and collectivism at the trait level (Markus and Kitayama, 1991). An independent self-construal means to construe oneself as a unique and separate entity from others, which results in a tendency for self-oriented behavior, where personal goals and values are prioritized. By contrast, an interdependent self-construal entails that interpersonal harmony and group cohesion are prioritized, resulting in behaviors motivated by social adjustment and concessions. The construct of interdependence was further refined to not only distinguish between cultures, but also between gender (Kashima et al., 1995; Cross and Madson, 1997). Specifically, women were argued to be higher in *relational* interdependence compared to men, which refers to the emotional connection and feeling of relatedness with intimate others such a family members, partners, or close friends (Cross et al., 2000; Maddux and Brewer, 2005). By contrast, *collective* interdependence reflects the level of connectedness to a larger group with a shared identity, such as one’s extended family, village, or country (Kashima and Hardie, 2000). While females have been proposed to be higher in relational interdependence, others have proposed that males in turn are higher in collective interdependence (Baumeister and Sommer, 1997; Gabriel and Gardner, 1999; Maddux and Brewer, 2005).

To further understand the effect of self-construal on psychological processes, relying on between-group comparisons may be insufficient (Oyserman et al., 2002). Importantly, self-construal is not a fixed trait, but can be seen as a dynamic mindset, that interacts with the social situation (Triandis, 1994; Ybarra and Trafimow, 1998; Oyserman and Lee, 2008). In certain situations, behavior and motivation could be guided relatively more by independent concepts (e.g., uniqueness, self-assertion, personal mastery), whereas in other situations they could be guided more by interdependent values and goals (e.g., concern for interpersonal harmony, conflict avoidance) (Triandis, 1995). An effective way of studying self-construal therefore is using self-construal priming, which activates a semantic network at the subconscious level (Oyserman et al., 2014). Indeed, a plethora of research has established that making independent and interdependent concepts salient activates cognitive schemas, which subsequently affect social cognition and behavior (Kühnen et al., 2001; Oyserman and Lee, 2008).

Recently, a study by Flinkenflogel et al. (2017) found that fairness considerations of females and males were differentially affected by self-construal priming, when presented with an unequal division in the Ultimatum Game (UG). The UG is a two-player social dilemma where a proposer offers a division of a sum, and the responder can subsequently choose to either accept or reject that offer (Güth et al., 1982). The prime entailed a modified version of the university’s mission statement which emphasized either independent or interdependent social norms, such as thinking about either own or shared goals, respectively. They found that when primed with interdependence, females rejected the offer on average less compared to when primed with independence; males however, displayed the reverse pattern. This suggests that making selective aspects of self-construal salient may interact with gender differences, either in default self-construal or in other characteristics associated with gender.

This aligns with previous studies that have found interactions between self-construal and gender in social behavior (Gabriel and Gardner, 1999; Maddux and Brewer, 2005), and may also be relevant to the large body of research on gender differences in fairness (Eckel and Grossman, 2008; Croson and Gneezy, 2009; Ergun et al., 2012; Espinosa and Kovářík, 2015), which have yielded inconsistent results so far (Dulebohn et al., 2016). Accordingly, meta-analyses have failed to establish whether either gender is more cooperative, altruistic, or fair (Balliet et al., 2011; Engel, 2011; Güth and Kocher, 2014). Possibly, situational characteristics interact with the self-construal of males and females, leading to differential effects on fairness. For instance, males can display less cooperative behavior compared to females when there is no tangible connection with the other person, while being relatively more cooperative with someone from a group with a shared identity (Van Vugt et al., 2007). As a result, subtle variations in the experimental setup might result in different outcomes for each gender when assessing fairness considerations, despite using similar measures (Croson and Gneezy, 2009; Espinosa and Kovářík, 2015).

Examining neural mechanisms may help to provide more insight in the motivations underlying decision-making, such as the interplay between norms, reward, and perspective-taking in social cognition (Rilling and Sanfey, 2011; Ruff and Fehr, 2014). Social dilemmas as the UG have been successfully employed to identify the neural components underlying social decision-making (e.g., Sanfey, 2007; Tabibnia et al., 2008; Gospic et al., 2011; Civai et al., 2012). For instance, there is a substantial body of evidence that consistently demonstrates that activity in the anterior insula (AI) correlates with an increased likelihood of the rejection of unfair offers (Gabay et al., 2014; Feng et al., 2015). Following their landmark research, Sanfey et al. (2003) proposed that AI activity accordingly represented the initial negative emotional reaction in response to unfair offers. This notion has been further refined by research showing increased AI activity in response to offers originating from both human and computers (Sanfey et al., 2003), as well as when subjects made decisions for themselves or third parties (Civai et al., 2012; Corradi-Dell’Acqua et al., 2012). By contrast, activity in the ventromedial prefrontal cortex (vmPFC), has only been reported when subjects made decisions for themselves (Civai et al., 2012; Corradi-Dell’Acqua et al., 2012). This area has been connected to emotional components of social decision-making (Rilling and Sanfey, 2011) as well as theory of mind and self-referential processing (McCabe et al., 2001; Rilling et al., 2004; Bhatt and Camerer, 2005).

Accordingly, the AI has been ascribed a general role in signaling norm violations, rather than the emotional response to unfairness (Klucharev et al., 2009; Zinchenko and Arsalidou, 2017). Indeed, this was exemplified by a recent study which found that AI activity was related to the expected height of the offer (Cheng et al., 2017). By informing how much other respondents had received, the authors manipulated the expectation of the participant. In this case, both unequal offers as well as lower than expected offers were associated with greater AI activity, which were explained as norm violations. Activation of the vmPFC in turn seems to represent the evaluation of decision-making that affects the self within a social context (Civai et al., 2012; Corradi-Dell’Acqua et al., 2012). For instance, Grecucci et al. (2012) found that downregulating negative emotions by reappraising the proposer’s intent primarily affected vmPFC activity during unfair offers. In addition, increased vmPFC activity was observed when the subject’s decision conflicted with that of the group (Wei et al., 2013). Furthermore, recent studies have found greater activity in the vmPFC for females compared to males in response to unfair offers, even in the absence of behavioral differences (Dulebohn et al., 2016; Kopsida et al., 2016). The increased activity was hypothesized to reflect female’s adaptation of behavior in order to reject unfair offers, reflecting underlying gender differences in social decision-making (Kopsida et al., 2016).

In the current study, we investigated the relationship between self-construal and gender differences on fairness considerations by using priming methodology. To prime self-construal, we used the Mission prime employed by Flinkenflogel et al. (2017), which emphasizes either independent or interdependent norms within the context of a shared identity (i.e., belonging to the student body). Prior research suggests that the effects of social norms become more pronounced when group identity is made salient (Glynn, 1997; Hogg and Reid, 2006; Rimal and Lapinski, 2015). Based on a previous study, we predicted that emphasizing interdependent values to females would result in lower rejection rates in response to unfair offers, as females tend to construct a more relational interdependent self (Flinkenflogel et al., 2017). Relational interdependence has been associated with taking perspective of others and harmony-oriented behavior (Cross and Madson, 1997; Cross et al., 2000). As males generally construct a more collective interdependent self, we expected relatively higher rejection rates when interdependence is brought to mind, as an unfair offer is perceived as a violation of the prescribed social norm to be fair. In the brain, we expected that this would be reflected in reduced AI and vmPFC activity in the interdependent condition compared to the independent condition for females, as the AI and vmPFC have been attributed with a central role in the processing of social norms and self-referential behavior in the UG (Rilling and Sanfey, 2011; Ruff and Fehr, 2014; Zinchenko and Arsalidou, 2017), and the consequential rejection of unfair offers. For the male participants in turn we expected the reverse; higher activity in the AI and vmPFC in the interdependent compared to the independent condition. Finally, we expected the effect of the prime to supersede that of trait self-construal, but added a trait self-construal measure as control.

## Materials and Methods

### Participants

One hundred healthy right-handed subjects between the ages of 18 and 28 (*M* = 20.96; *SD* = 2.15) participated in the study, balanced over gender (50 males, 50 females). Participants were recruited on campus of the University of Amsterdam (UvA) and VU University Amsterdam (VUA). All participants were native Dutch speakers and provided written informed consent. They were paid for participation, and received a bonus based on a random selection of their choices in the UG. Two participants were excluded from the behavioral data analysis due to missing or incomplete data. One participant was removed due to excessive head movement (>2 mm). The final dataset included 97 participants, divided between 48 participants in the independent-mindset condition (*M*_age_ = 20.88, *SD*_age_ = 2.02; 24 males), and 49 in the interdependent-mindset condition (*M*_age_ = 21.08, *SD*_age_ = 1.49; 24 males). Procedures were approved by the ethical committees of the Faculty of Behavioral and Movement Sciences of the VUA, as well as the Spinoza fMRI Scanning center.

### Design

#### Trait Self-Construal Measures

To measure trait self-construal, we used a modified version of the Oyserman (1993) scale, translated to Dutch. The scale consisted of a total of twenty-two items; eleven items relating to trait independence (e.g., “I don’t care what other people think about me, as long as I am happy”; α = 0.78) and eleven items measuring trait interdependence (e.g., “Relationships with others are more important than my own accomplishments”; α = 0.71). To construct a singular factor for analysis, a ratio score of independence and interdependence scores was computed by subtracting the independent score from the interdependent score, and dividing by the independent score (TSC) (Flinkenflogel et al., 2017). A higher score on TSC indicated a higher ratio of interdependence relative to independence.

#### Ultimatum Game

The UG is a two-player social dilemma where one person proposes a division of a sum (e.g., 100), and a responder can either accept or reject that proposal. However, if the proposer decides to reject, neither gets paid. In the current study, participants were placed in the responder role throughout the experiment. Offers were presented in the full range between 10 and 50 (e.g., 37 for the participant, 63 for the proposer) to create sufficient variation in order to avoid repetitiveness of recurring offers.

**FIGURE 1:**
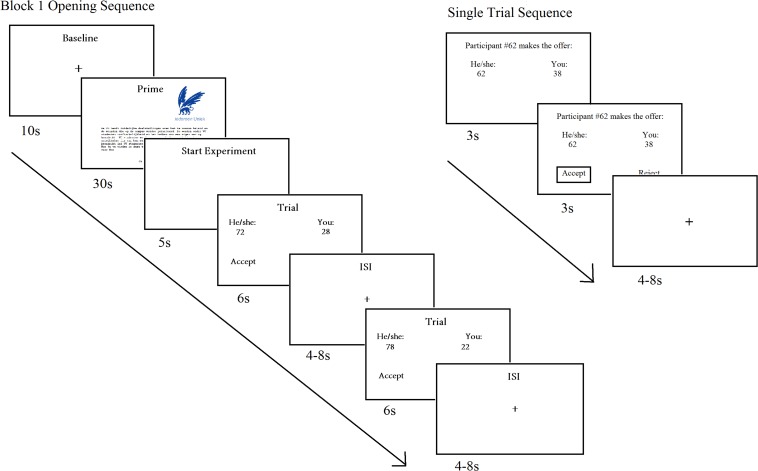
Trial sequence of the Ultimatum Game offer. Participants commenced with a baseline scan, followed the self-construal prime, and a 5 s pause before the experimental trials. Each experimental trial was followed by a variable interstimulus interval, and experimental and control trials were interlaced. The control trial sequence was identical to the experimental trial.

They were briefed they would receive offers from previous participants, and the outcome of their decisions would affect both themselves and the proposers. In reality, the proposals were preprogrammed and randomized in advance, but presented in similar order across participants. To increase believability, participants were told they would be provided with the opportunity to propose ten divisions for future participants after the experiment. The UG offer was presented at the beginning of each trial. After 3 s, a response option (accept/reject) was displayed below the offer for an additional 3 s. After participants made their decision, a box highlighted the selected option, which remained visible for the remainder of the 3 s. The trial ended with a jittered interstimulus interval presented as a fixation cross, which varied between 4 and 8 s (Figure 1).

Control trials consisted of a similar sequence, with the exception that participants could choose from two presented numbers for a free win, and the choice options to accept or reject were replaced with “left” or “right.” While the numbers in the experimental trials always added up to 100 (equivalent to €10), the control trials did not. Participants played a total of 80 experimental trials, and 25 control trials. The experimental trials were balanced within each block between 13 and 14 “fair” offers (ranging between 36 and 50 for the participant, and the remainder of a total of 100 for the proposer), and 13–14 “unfair” offers (ranging between 10 and 35 for the participant, and the rest for the proposer). The cutoff point for fair and unfair offers was based on the consideration that 5–5 and 6–4 divisions are generally accepted by the vast majority of respondents. By contrast, offers from 7 to 3 and up are on average rejected by the majority of responders (more than 50%) (e.g., Henrich et al., 2001; Oosterbeek et al., 2004; Güth and Kocher, 2014). Since our range of offers is incremental in steps of 1 (e.g., 62–38, 73–27), we decided to place the cutoff in the middle of the 6–4 and 7–3 divisions, which is 65–35. In addition, we included a question in the questionnaire following the fMRI experiment which asked respondents what their own cutoff point was for rejecting. The mode response was 35, while the average was 33. This provided us with sufficient confidence in our classification of fair and unfair.

#### Self-Construal Priming Manipulation

The employed prime was the Mission prime described by Flinkenflogel et al. (2017). The prime consisted of the VUA mission statement, which was modified to represent either independent or interdependent values. In the independent-mindset condition the text read (translated from Dutch): “The VUA has a clear policy concerning the goals and values on campus. The VUA emphasizes that students should develop independence and having their own opinion. VUA students are encouraged to develop skills that make them a unique individual. Students should maintain personal goals.” In the interdependent-mindset condition the underlined words were replaced by: honesty, equality, social and interpersonal (underlining for clarification, not presented in the original text). In addition, we included the VUA logo in the top right corner, with a modified slogan resembling the original format reading “everyone unique” in the independent-mindset condition, and “everyone equal” in the interdependent-mindset condition. To encourage participants to actively read the statement, they were asked to indicate with a yes/no response whether they thought the statement was applicable to themselves. However, their response was not used for analysis since the prime is supposed to work implicitly by activating the constructs of independence or interdependence in the minds of the participants regardless of their adherence to the prescribed values (Kühnen et al., 2001).

### Procedure

All participants read the information material and instructions before the testing procedure, and provided written informed consent. Participants received earplugs to lower background noise, and were laid supine in the scanner, with padding for fixation. They were instructed to place their right hand on a three-button box, with two functional buttons (forefinger and middle finger) for the UG. Participants commenced with a structural scan for 10 min, during which they received instructions for the UG, followed by 10 practice trials. After completion, they proceeded with the three experimental blocks. Each experimental block started and ended with a baseline trial, where they viewed a blank screen for 10 s. In the first block, the participants subsequently viewed the prime for 30 s. The experiment then commenced with the experimental trials. Block 1 consisted of 26 experimental trials and 9 control trials, block 2 of 27 experimental trials and 8 control trials, and block 3 of 27 experimental trials and 8 control trials. Each experimental block lasted between 6 and 7 min. At the end of the block was a short break, wherein participants could indicate when they were ready to proceed. After the fMRI experiment, participants moved to a separate secluded area where they completed a computerized questionnaire with the trait self-construal scale, basic demographics, and qualitative questions concerning their choices and strategy in the UG, as well as their experience in the scanner. The experiment lasted a total of 40–45 min, including the questionnaire. Participants received an average bonus of €1.60 (ranging between €0.80 and €2.50 based on the earnings from ten randomly selected rounds of the UG, in addition to a fixed payment of €25 for the participation.

### fMRI Image Acquisition

fMRI data were obtained at the Spinoza Center Amsterdam, using a 3.0 T Philips Achieva whole body scanner (Philips Healthcare, Best, Netherlands) equipped with a 32 channel head coil. A T2^∗^ EPI sequence (TR = 2 s, TE = 27.63 ms, FA = 76.1°, FOV 240 mm, voxel size 3 × 3 × 3 mm, 37 slices, 0.3 mm gap) was used, resulting in 240 images in Block 1, 225 images in Block 2, and 231 images in Block 3 (the first block was longer due to the priming manipulation, while the last was slightly longer due to the closure of the experiment). A T1-weighed anatomical scan was acquired for anatomical reference (TR = 8.2 ms, TE = 3.8 ms, FA = 8°, FOV 240 × 188 mm, voxel size 1 × 1 × 1 mm, 220 slices).

### Data Analysis

#### Behavioral Data

Behavioral data was analyzed in IBM SPSS Statistics 24. To examine whether there were differences between participants in TSC between gender or the priming conditions, we first performed a two-way ANOVA with gender and condition as factors, and TSC as the dependent variable.

To analyze the behavioral data, we calculated the average rejection rate per participant over all trials and blocks, and performed a two-way ANOVA with gender and condition as categorical independent variables, TSC as continuous independent variable, and all two- and three-way interaction effects. The significance level was set at α ≤ 0.05 throughout.

#### fMRI Data

Imaging data were reconstructed and analyzed using Statistical Parametric Mapping 12^1^ in MATLAB. Preprocessing of functional images for each participant included 3D motion correction using iterative rigid body realignment to minimize the residual sum of squares between the first and following functional scans. Next, rigid body coregistration to corresponding individual T1 images was performed using mutual information optimization, followed by spatial normalization into a common space, defined by the Montreal Neurological Institute (MNI) 152 T1 image (voxel size = 2 × 2 × 2 mm) template. Finally, data were smoothed by an 8 mm full width at half maximum (FWHM) Gaussian kernel. Slice timing correction was not performed. For every participant, a general linear model (GLM) was used to construct individual time courses for the onset of trial presentations of the experimental trials (accept and reject), as well as the control and baseline trials for all three runs separately. Because of habituation effects (see below), another GLM was constructed with just the first run. Finally, the 6 realignment parameters were included, resulting in a total of 10 regressors in the full model. We modeled a trial length of 3 s, the presentation time of the offer. In the experimental trials a distinction was made between the accepted and rejected trials, which were contrasted against each other in the first-level analysis (reject > accept). For group analyses, we constructed a full-factorial model with the two contrasts of gender and condition.

In addition, we performed a head motion analysis to assess whether there were differences between gender and condition on the 6 direction variables. While there was no effect of condition, *F*(1,58) = 0.019, *p* < 0.892, there was a significant difference between gender: *F*(1,58) = 15.061, *p* < 0.001, η^2^ = 0.21. Accordingly, we performed a *post hoc* analysis to analyze the individual parameters. Following a Bonferroni correction, the alpha level was adjusted to α ≤ 0.008. The analysis revealed that females displayed significantly more movement on the *Z*-axis, *B* = 0.150, *SE* = 0.049, *t*(58) = 3.084, *p* = 0.003, 95% CI [0.053, 0.247]. There were no significant effects on the *X*-axis (*B* = 0.01, *SE* = 0.016, *t*(58) = –0.025, *p* = 0.980, 95% CI [–0.032, 0.032]), *Y*-axis (*B* = 0.051, *SE* = 0.026, *t*(58) = 1.952, *p* = 0.056, 95% CI [–0.001, 0.103]), pitch (*B* = –0.01, *SE* = 0.001, *t*(58) = –1.082, *p* = 0.284, 95% CI [–0.003, 0.001]), roll (*B* = 0.001, *SE* = 0.001, *t*(58) = 0.138, *p* = 0.891, 95% CI [–0.001, 0.001]), or yaw (*B* = 0.001, *SE* = 0.001, *t*(58) = –0.194, *p* = 0.847, 95% CI [–0.001, 0.001]).

A whole brain analysis was performed to identify general patterns of rejection over acceptance, with the whole-brain threshold set at *p* < 0.001 uncorrected. Effects were considered significant at α ≤ 0.05 FWE cluster-level correction. Significant clusters were interpreted using the AAL-atlas. Second, an a priori voxel-based Region of Interest (ROI) analysis based on Small Volume Correction (SVC) was performed to investigate regions involved in social decision-making and self- and other-representation in the UG. We used peak coordinates with a sphere of 10 mm around the left and right insula as reported in a meta-analysis on UG behavior in imaging studies (Gabay et al., 2014), while the vmPFC was highlighted as region of interest for gender differences in the UG in a neuroimaging study by Kopsida et al. (2016, see Supplementary Table S4). While Dulebohn et al. (2016) similarly reported the vmPFC as a region of interest for gender differences in the UG, we were unable to uncover the specific coordinates. Corrected *p*-values are reported at a significance level of α ≤ 0.05 FWE cluster level corrected (either whole-brain or after SVC with above-mentioned ROIs), after thresholding at an initial whole-brain *p* < 0.001 uncorrected threshold. Upon publication of the manuscript, all contrast maps will be uploaded to NeuroVault^2^ to allow other researchers to interactively vary the threshold of the T-maps and compare subthreshold results to their hypotheses.

## Results

### Behavioral Analysis

**FIGURE 2:**
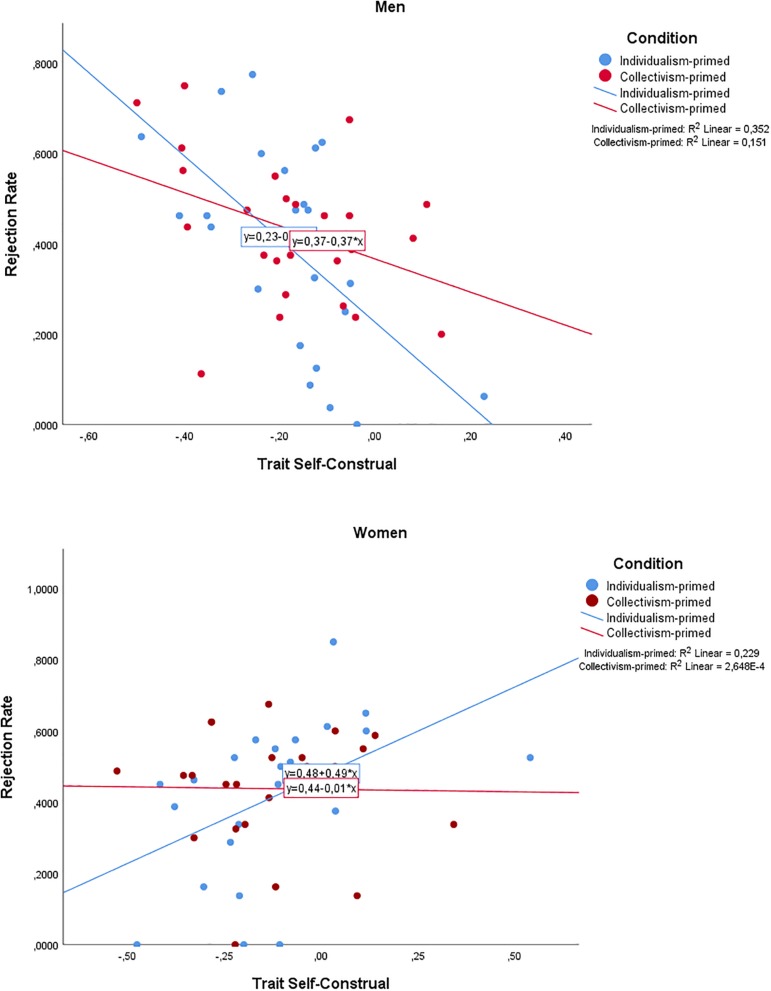
Three-way interaction between Gender, Trait Self-Construal (TSC), and Priming Conditions broken down by Gender. For independent-primed men, a *lower* value of TSC resulted in higher rejection. For women, a higher value of TSC resulted in greater rejection rates. There were no differences in the collectivistic priming condition. TSC is mean-centered.

First an ANOVA was conducted to examine whether there were gender differences in TSC between gender or condition. While females reported a slightly higher ratio of interdependence (*M* = –0.13, *SD* = 0.03) relative to males (*M* = –0.18, *SD* = 0.03), the difference was not statistically significant, *F*(1,96) = 2.169, *p* = 0.144. Furthermore, there was no significant difference between the participant group primed with independence (*M* = –0.15, *SD* = 0.03) and interdependence (*M* = –0.15, *SD* = 0.03), *F*(1,95) = 0.007, *p* = 0.935. There was no difference in base rejection rates between males (*M* = 0.41, *SD* = 0.19) and females (*M* = 0.43, *SD* = 0.19), *F*(1,95) = 0.003, *p* = 0.957. The difference between the independent (*M* = 0.41, *SD* = 0.22) and interdependent (*M* = 0.43, *SD* = 0.16) condition was also not significant, *F*(1,90) = 1.056, *p* = 0.307. In order to investigate rejection behavior in the UG, a two-way ANOVA was conducted with gender and condition as categorical independent variables, TSC as continuous independent variable, and all two- and three-way interaction effects, with the average rejection rate as dependent variable. The outcome of rejection rate was qualified by a significant three-way interaction between gender, condition, and TSC: *F*(1,90) = 6.519, *p* = 0.012, η^2^ = 0.68 (Figure 2). While neither the condition by TSC interaction was significant, *F*(1,90) = 0.11, *p* = 0.916; nor the condition by gender interaction, *F*(1,90) = 3.385, *p* = 0.069; the interaction between gender and TSC was, *F*(1,90) = 18.059, *p* = 0.001, η^2^ = 0.167. Further contrasts specifically for each gender (i.e., looking into the TSC × priming condition interaction in the male and female group separately) revealed that this interaction was non-significant for both men [*F*(1,46) = 3.141, *p* = 0.083], and women [*F*(1,44) = 3.597, *p* = 0.064]. However, for men in the individualist priming condition, a lower value of TSC resulted in higher rejection rates (*B* = –0.919, *SE* = 0.239, *t*(46) = –3.850, *p* < 0.001), whereas for women in the individualist priming condition, a higher TSC value resulted in higher rejection rates (*B* = 0.495, *SE* = 0.180, *t*(44) = 2.750, *p* = 0.009). In the collectivist priming condition, there was no relationship between TSC and rejection rate (for men, *B* = –0.366, *SE* = 0.201, *t*(46) = –1.827, *p* = 0.074; for women *B* = –0.014, *SE* = 0.199, *t*(44) = –0.070, *p* = 0.944).

### fMRI Analysis

Analysis of the response contrast (reject > accept) on whole brain yielded higher activation in the left fusiform gyrus (*p* = 0.004), right inferior parietal lobe (*p* < 0.001), right middle frontal gyrus (*p* < 0.001), right dorsolateral prefrontal cortex (*p* = 0.001), left inferior parietal lobe (*p* = 0.013), left parahippocampus (*p* = 0.009), right visual association area (*p* < 0.001), and the extrastriate cortex (*p* = 0.001) (Table 1), which were consistent with reported regions in the meta-analysis by Gabay et al. (2014). However, the robust main effects in the AI, our primary region of interest, was not replicated. We then performed the separate analyses on the condition by gender interaction, and individual gender and condition contrasts, but did not establish any significant effects in the AI or vmPFC. Subsequent SVC analyses on the hypothesized regions of interests equally did not uncover any significant findings.

**TABLE 1:** Significant activation clusters for the whole brain analysis of reject > accept.

**Brain region**	**HEM**	***X***	***Y***	***Z***	**Voxels**	***F*-value**
Fusiform gyrus	L	–24	–76	–10	448	87.31
Visual association area	R	–22	–72	–8	760	63.62
Extrastriate cortex	R	28	–78	20	564	36.80
Inferior parietal lobe	R	56	–36	52	2080	32.72
Middle frontal gyrus	R	32	14	54	946	30.59
Dorsolateral prefrontal cortex	R	40	34	20	1138	29.32
Inferior parietal lobe	L	–46	–44	44	339	24.57
Parahippocampus	L	–26	–30	–16	366	22.63

*Coordinates (mm) are in MNI space. HEM = hemisphere. All clusters reported survived the family-wise error (FWE) correction *p* < 0.05 with an extend threshold of 10 voxels.*

### Follow-Up Analysis

Previous literature suggests that habituation effects occur in long, repetitive experimental setups, resulting in decreased neural activation (Delgado et al., 2005; Dulebohn et al., 2016). As we suspected this occurred in the current study, we performed a separate habituation analysis with the response contrast at first level (reject > accept), and the difference in activation between the first and last block at second level (block 1 > block 3). This analysis revealed a large cluster of activity in the left insula [*p*(FWE) = 0.03, T = 4.07, 326 voxels, MNI coordinates (*x, y, z*): –40, 4, 2]. Contrasting the third block with the first in turn primarily revealed greater activation in both the right visual cortex [*p*(FWE) = 0.001, T = 5.07, 739 voxels, MNI coordinates (*x, y, z*): –14, –86, 2], as well as the left visual cortex [*p*(FWE) = 0.002, T = 4.70, 643 voxels, MNI coordinates (*x, y, z*): –26, –86, –6], indicating participants were mostly processing the offers on a visual level during the later blocks.

Since this analysis suggested marked habituation effects we performed a follow-up analysis of the data of the first block separately, as is a common strategy (Berns and Bell, 2012; Hunt et al., 2012; Brown et al., 2014; Dulebohn et al., 2016). To ensure a reliable analysis of the BOLD signal, we used the subset of participants that both accepted and rejected at least 1/3 of the total amount of trials in that block (8 trials for each type) based on a recently published fMRI study using a social dilemma task (Lemmers-Jansen et al., 2018). Accordingly, 38 participants were discarded from analysis, due to insufficient number of accepted or rejected trials. The 59 participants included in the dataset for fMRI analysis were divided as follows: 25 participants in the independent-mindset condition (*M*_age_ = 20.74, *SD*_age_ = 1.85; 10 males), and 34 in the interdependent-mindset condition (*M*_age_ = 21.50, *SD*_age_ = 1.96; 16 males). The mean rejection rate in the first block was 42% (SD = 20%), which amounted to a mean of 10.9 rejected trials per participant. Within this group there were no significant results at the behavioral level.

The main effect of the response contrast (reject > accept) yielded activation in the left AI on whole brain level [*p*(FWE) = 0.007, T = 4.38, 395 voxels, MNI coordinates (*x, y, z*): –52, 8, –2] (when controlling for TSC [*p*(FWE) = 0.008, T = 4.33, 378 voxels, MNI coordinates (*x, y, z*): –52, 8, –2]. The right AI equally displayed increased activity, but this was only significant after lowering the height threshold to *p* = 0.005, [*p*(FWE) = 0.021, T = 3.94, 124 voxels, MNI coordinates (*x, y, z*): 58, –22, 16], when controlling for TSC the right AI activity remained precisely the same.

**FIGURE 3:**
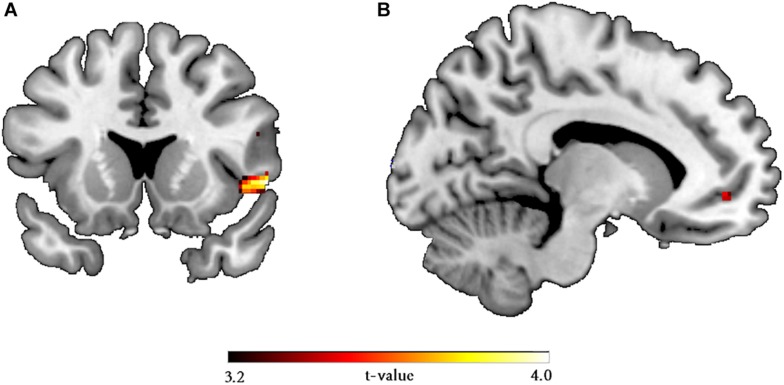
Whole brain statistical maps displaying the **(A)** independent versus interdependent priming contrasts, with greater activation in the AI for the independent mindset condition, and **(B)** female versus male gender contrast, with greater activation in the vmPFC for females, which was mediated by trait self-construal.

Furthermore, compared to interdependent-primed participants, the independent-primed participants showed greater activity in the right AI for the reject versus accept contrast [*p*(FWE) = 0.021, T = 3.75, 14 voxels, MNI coordinates (*x, y, z*): 46, 14, –4, SVC insular mask taken from Gabay et al. (2014)]. When controlling for TSC, the significance of the insular activities modulated by the self-construal prime did not change [*p*(FWE) = 0.023, T = 3.71, 11 voxels, MNI coordinates (*x, y, z*): 46, 14, –4], compared to the model where TSC was not included. Reversing the contrast (interdependent > independent) revealed no effects. In addition, we found more activity in the left vmPFC for females compared to males [*p*(FWE) = 0.038, T = 3.27, 1 voxel, MNI coordinates (*x, y, z*): –16, 48, –6, SVC within a vmPFC mask taken from Kopsida et al. (2016)] for the reject versus accept contrast (Figure 3). When including TSC in the model, we founded decreased activity (to non-significant level) in the left vmPFC modulated by the gender differences compared to when not controlling for TSC, suggesting that the gender effect was not robust. There was no significant activity for the reverse contrast (males > females). Finally, there were no significant interactions throughout.

As the head motion analysis had revealed a significant difference between gender on the *Z*-axis, we performed a Pearson’s correlation to assess whether the established effect was possibly due to head motion. However, there was no significant correlation, *r* = –0.052, *n* = 59, *p* = 0.697. As the gender difference in the vmPFC was hypothesized beforehand based on prior literature (Dulebohn et al., 2016; Kopsida et al., 2016), it seems likely that the found effect in the vmPFC is due to the experimental manipulation and not head motion differences.

## Discussion

In the current study we used priming methodology to investigate the underlying neural effects of self-construal on fairness considerations. Based on a previous study, we predicted opposing effects of the self-construal prime for each gender; females would reject relatively more offers when primed with independence, while males would reject more often when primed with interdependence. We expected higher rejection rates to be mirrored by AI and vmPFC activity. The analysis revealed, among others, greater activity in the right dorsolateral prefrontal cortex, right middle frontal gyrus, left fusiform gyrus, and the left inferior parietal lobe for the response contrast (reject > accept), which were consistent with previous findings (Gabay et al., 2014). However, we found no support for the main predictions concerning the gender by self-construal priming interaction for AI and vmPFC activity at the neural level, nor did we replicate our previous findings at the behavioral level.

At the behavioral level, we observed a significant three-way interaction between priming condition, gender and trait self-construal. Follow-up analyses indicated that rejection rate was driven by a trend level interaction between trait self-construal and priming condition which differed between genders. In the individualist priming condition, males with lower levels of interdependence relative to independence, had a higher rejection rate, whereas the opposite – higher rejection rates with higher levels of interdependence to independence, was found in females. No relationship between trait self-construal and rejection rate was found in the collectivist priming condition. These results suggest that priming effects may vary dependent on individual characteristics of the participant. This is in line with previous reflections on priming methodology pointing out that priming effects are especially sensitive to variations in experimental settings and participant populations (Cesario, 2014; Loersch and Payne, 2014). It should be noted that the predicted interaction between self-construal priming and gender in the previous study was consistent over three different locations, including both laboratory and outside settings, as well as hypothetical and monetary payouts (Flinkenflogel et al., 2017). However, in our previous studies, participants were primed in quiet, and relatively more familiar settings. In the current study, the majority of participants were being scanned for the first time, which is a loud, somewhat claustrophobic, and therefore occasionally overwhelming experience. These aspects of the experimental setting may have impacted on the behavioral effects of the prime.

It is further possible that the prime affected the subconscious appraisal of the offers, but was not strong enough to be translated into behavioral action as this involves multiple steps. According to the semantic-procedure model, which outlines how self-construal priming affects behavior, concepts are represented by a network of pre-existing knowledge (Kühnen et al., 2001). Self-construal priming draws on these concepts in long-term memory to activate a procedural mode of thinking. Procedures in turn shape the corresponding cognitive style to process relevant information for fairness considerations (Oyserman and Lee, 2008). Finally, the effect of self-construal priming needs to reach a threshold to be translated into overt behavior, in this case the dichotomous decision to either accept or reject. Taken together, it is possible that the prime activated implicit schemas that affected the subconscious appraisal of the offers, but could not be evidenced in behavioral responses.

Upon closer inspection of the fMRI data, further analysis revealed a habituation effect of neural activity in the key regions of interest. Previous findings have demonstrated that diminished activity occurs in response to aversive stimuli (e.g., Phelps et al., 2001). In addition, it is likely that the repetitive nature of the task led to inattention (Hunt et al., 2012). This was confirmed by analyses comparing the first to the last block, which showed diminished activity in brain regions related to the UG. Subsequently, we performed a follow-up analysis with a subsample of participants limited to the first block (Delgado et al., 2005; Dulebohn et al., 2016). The analysis revealed heightened activity in the left AI for the response contrast (reject > accept) at the whole brain level, which replicated the robust finding that heightened AI activity is associated with rejected offers. Furthermore, we found that priming interdependence resulted in reduced AI activity compared to the independent condition. Finally, females displayed higher levels of vmPFC activity than males, although this gender difference disappeared after controlling for trait self-construal.

These follow-up results suggest that self-construal priming affects neural components of decision-making, in line with prior research suggesting cultural influences can modify AI activity (Han and Northoff, 2008; Immordino-Yang et al., 2014). The attenuated AI activity in the interdependent compared to the independent condition may reflect a lessened negative emotional response to unfair offers (Sanfey et al., 2003). This was theorized to result from motivations as conflict avoidance and harmony maintenance, which are associated with interdependence (Triandis, 2001). The AI has been implicated in a wide range of mental states including negative emotions as disgust and pain (Wiech and Tracey, 2009; Wager and Barrett, 2017), as well as risk prediction (Preuschoff et al., 2008), giving rise to the possibility that it contributes to learning from negative social interactions to guide future behavior (Rilling et al., 2008).

Alternatively, it might indicate that in the interdependent condition the unfair offers are cognitively regarded as less unfair, suggesting that self-construal modulates the way the offer is perceived in terms of norm expectancy. Recent advancements in neuroscience have highlighted the role of the AI in social norms; specifically, a meta-analysis found that norm violation was most strongly correlated with AI activity (Zinchenko and Arsalidou, 2017). This notion is supported by a recent study which manipulated the expectancy of the offer prior to the proposal (Cheng et al., 2017). The authors found increased AI activity for lower than expected offers irrespective of the proportion of the offer itself, suggesting AI activity does not necessarily reflect an emotional reaction, but rather a heuristic signaling the violation of social norms (Klucharev et al., 2009; Zinchenko and Arsalidou, 2017). As the Mission prime prescribes social norms from the context of a shared identity, it is likely that the modified AI activity reflects differential norm processing in the separate conditions. However, since AI activity was not mirrored by rejection rates in the behavioral data, these interpretations are speculative in nature, and need to be interpreted with caution.

In addition, the follow-up analysis revealed greater left vmPFC activity for unfair offers for females compared to males, which corresponds with previous studies investigating gender differences in the UG (Dulebohn et al., 2016; Kopsida et al., 2016). The increased activity in the vmPFC can be interpreted as greater activation of the intuitive-emotional system of decision-making in response to unfair offers (Sanfey et al., 2006; Feng et al., 2015), which has been explained to reflect a stronger reaction of females to unfairness (Dulebohn et al., 2016). In addition, burgeoning research from cultural psychology has allocated a central role of the vmPFC in self-referential processing (Harada et al., 2010; Kitayama and Park, 2010; Han et al., 2013). For instance, by using priming methodology Zhu et al. (2007) found that judging traits of their own mothers relative to that of others activated vmPFC in Chinese participants, who represented a predominantly interdependent self-concept, but not in Western subjects, who represented a predominantly independent self-concept. Similarly, priming independence increased the neural differentiation of the self with their mother and an unidentified other in the vmPFC in bicultural participants, whereas priming interdependence decreased the differentiation between the self and others (Ng et al., 2010). In addition, Chiao et al. (2010) showed that priming independent and interdependent self-construals while thinking of oneself in different social contexts is reflected by variations in vmPFC activity.

Furthermore, we found that the effect of gender on vmPFC activity decreased when controlling for trait self-construal, which suggests that the gender effect is mostly driven by differences in trait self-construal which, in our data, differed (albeit, non-statistically) between gender (although previous studies have consistently reported gender differences in self-construal, e.g., Gabriel and Gardner, 1999; Maddux and Brewer, 2005; Flinkenflogel et al., 2017). This coincides with the suggestion that females generally construct a more interdependent self-compared to males (Cross and Madson, 1997). It is therefore possible that the initial heightened vmPFC activity displayed by females was indicative of differential self-referential processing, which signifies a greater role of other-processing during social decision-making.

Finally, although there were no differences in rejection rate between gender, the outcome of the decision was driven by an interaction between gender, the priming conditions and trait self-construal: when primed with independence, males rejected more often when relatively low in trait interdependence, while females rejected more with *higher* reported levels of trait interdependence. No differences occurred when primed with interdependence. This tentatively suggests that social decision-making is at least partially affected by an underlying role of trait self-construal, which is manifested in different ways depending on gender.

### Limitations

The primary drawback of the current study was the encountered habituation effect of the repetitive UG trials, which was suggested by the lack of insula activity in response to rejected offers in the second and third blocks. This limited us to a follow-up analysis of the first block, which prohibited a complete investigation of the effects of gender and priming. Other researchers have attempted to counter this effect by displaying pictures of the proposer (e.g., Tabibnia et al., 2008; Cheng et al., 2017) or recording videos of proposals (e.g., Gospic et al., 2011; Kopsida et al., 2016). However, we opted against presenting visual imagery, as it is likely to elicit a wider range of activity, including affective bias toward the proposer. Furthermore, there are sufficient reports of experiments using similar setups as the current study (Kirk et al., 2011, 2016; Civai et al., 2012; Corradi-Dell’Acqua et al., 2012; Wei et al., 2013). Therefore, it is unclear which factors lead to an occurrence of the habituation effect. One possibility is that the habituation effect occurs more regularly, but is less visible due to publication bias (Jennings and Van Horn, 2012; Ioannidis et al., 2014). Even so, the current results suggest that future studies should take such effects into account. The research should also be interpreted in the light of the fact that our sample consisted of students, which have been argued to lack representativeness of a general population, both nationally and internationally (Henrich et al., 2010). For instance, college students’ cultural values might differ from those of the remainder of the population because of, for example, a higher socioeconomic background (Hanel and Vione, 2016). Recent research contented that socioeconomic differences could also produce group differences that mimic the commonly found differences between independent and interdependent self-construals (Cohen and Varnum, 2016).

Furthermore, research specifically dedicated to investigating the neural effects of priming found that activating both independent and interdependent mindsets increases vmPFC activity (Wang et al., 2013). The vmPFC therefore seems to be intimately involved in self-referential processing, regardless of the specific priming condition. It is therefore possible that both the priming conditions in the current experiment increased activity in the vmPFC, which nullified respective differences between the conditions when contrasted. It is possible that this effect would become clear when including a baseline (neutral) condition.

## Conclusion

In the current study we examined the neural correlates of self-construal and gender on fairness considerations, using priming methodology. Our prediction that priming interdependence would result in higher rejection rates for males, but lower rejection rates for females, was not supported. However, when we limited our analysis to the first block, a follow-up analysis confirmed the effect of heightened activity in the AI in response to rejected offers, and revealed some promising findings for future research.

The results tentatively suggest that salient self-construals influence the way fairness is processed at the neural level; priming an interdependent mindset resulted in reduced AI activity compared to an independent mindset. Possibly, the interdependent mindset affected the evaluation of social norms (Zinchenko and Arsalidou, 2017) by favoring motivations as harmony maintenance and conflict avoidance, which impacted on the emotional or cognitive evaluation of unfair offers. However, this effect occurred in the absence of behavioral differences. Furthermore, we found differential activity in the left vmPFC for females over males in line with previous research, elucidating gender differences in the decision-making process. These findings might help future research in elucidating the underlying mechanisms of the effect of self-construal on fairness considerations.

## Data Availability

The datasets generated for this study are available on request to the corresponding author.

## Ethics Statement

This study was approved by and according to the guidelines of the Ethical Committee of the Faculty of Behavioral and Movement Sciences of the Vrije Universiteit Amsterdam with written consent from all subjects. All subjects gave written informed consent in accordance with the Declaration of Helsinki. The protocol was approved by the Ethical Committee of the Faculty of Behavioral and Movement Sciences of the Vrije Universiteit.

## Author Contributions

NF, LK, and MK designed the study. NF executed the study. NF, MK and T-VV analyzed the data. NF and LK wrote the manuscript, while all authors provided input on the content and style of the manuscript. All authors discussed the results, reviewed the article, and gave final approval for the version to be published.

## Conflict of Interest Statement

The authors declare that the research was conducted in the absence of any commercial or financial relationships that could be construed as a potential conflict of interest.

## Footnotes

1 www.fil.ion.ucl.ac.uk/spm/

2 www.neurovault.com

## References

[B1] Balliet D. Li N. P. Macfarlan S. J. Van Vugt M. (2011). Sex differences in cooperation: a meta-analytic review of social dilemmas. *Psychol. Bull.* 137 881–909. 10.1037/a0025354 21910518

[B2] Baumeister R. F. Sommer K. L. (1997). What do men want? Gender differences and two spheres of belongingness: comment on Cross and Madson (1997). *Psychol. Bull.* 122 38–44. 10.1037//0033-2909.122.1.38 9204778

[B3] Berns G. S. Bell E. (2012). Striatal topography of probability and magnitude information for decisions under uncertainty. *Neuroimage* 59 3166–3172. 10.1016/j.neuroimage.2011.11.008 22100418 PMC3288668

[B4] Bhatt M. Camerer C. F. (2005). Self-referential thinking and equilibrium as states of mind in games: fMRI evidence. *Games Econ. Behav.* 52 424–459. 10.1016/j.geb.2005.03.007

[B5] Brown T. I. Whiteman A. S. Aselcioglu I. Stern C. E. (2014). Structural differences in hippocampal and prefrontal gray matter volume support flexible context-dependent navigation ability. *J. Neurosci.* 34 2314–2320. 10.1523/JNEUROSCI.2202-13.2014 24501370 PMC3913873

[B6] Buckholtz J. W. Marois R. (2012). The roots of modern justice: cognitive and neural foundations of social norms and their enforcement. *Nat. Neurosci.* 15 655–661. 10.1038/nn.3087 22534578

[B7] Cesario J. (2014). Priming, replication, and the hardest science. *Perspect. Psychol. Sci.* 9 40–48. 10.1177/1745691613513470 26173239

[B8] Cheng X. Zheng L. Li L. Zheng Y. Guo X. Yang G. (2017). Anterior insula signals inequalities in a modified Ultimatum Game. *Neuroscience* 348 126–134. 10.1016/j.neuroscience.2017.02.023 28223239

[B9] Chiao J. Y. Harada T. Komeda H. Li Z. Mano Y. Saito D. (2010). Dynamic cultural influences on neural representations of the self. *J. Cogn. Neurosci.* 22 1–11. 10.1162/jocn.2009.21192 19199421

[B10] Civai C. Crescentini C. Rustichini A. Rumiati R. I. (2012). Equality versus self-interest in the brain: differential roles of anterior insula and medial prefrontal cortex. *Neuroimage* 62 102–112. 10.1016/j.neuroimage.2012.04.037 22548807

[B11] Cohen A. B. Varnum M. E. (2016). Beyond East vs. West: social class, region, and religion as forms of culture. *Curr. Opin. Psychol.* 8 5–9. 10.1016/j.copsyc.2015.09.006 29506803

[B12] Corradi-Dell’Acqua C. Civai C. Rumiati R. I. Fink G. R. (2012). Disentangling self-and fairness-related neural mechanisms involved in the ultimatum game: an fMRI study. *Soc. Cogn. Affect. Neurosci.* 8 424–431. 10.1093/scan/nss014 22287263 PMC3624953

[B13] Croson R. Gneezy U. (2009). Gender differences in preferences. *J. Econ. Lit.* 47 448–474.

[B14] Cross S. E. Bacon P. L. Morris M. L. (2000). The relational-interdependent self-construal and relationships. *J. Pers. Soc. Psychol.* 78 791–808. 10.1037/0022-3514.78.4.191 10794381

[B15] Cross S. E. Hardin E. E. Gercek-Swing B. (2011). The what, how, why, and where of self-construal. *Pers. Soc. Psychol. Rev.* 15 142–179. 10.1177/1088868310373752 20716643

[B16] Cross S. E. Madson L. (1997). Models of the self: self-construals and gender. *Psychol. Bull.* 122 5–37. 10.1037/0033-2909.122.1.5 9204777

[B17] Decety J. Yoder K. J. (2017). The emerging social neuroscience of justice motivation. *Trends Cogn. Sci.* 21 6–14. 10.1016/j.tics.2016.10.008 27865787

[B18] Delgado M. R. Miller M. M. Inati S. Phelps E. A. (2005). An fMRI study of reward-related probability learning. *Neuroimage* 24 862–873. 10.1016/j.neuroimage.2004.10.002 15652321

[B19] Dulebohn J. H. Davison R. B. Lee S. A. Conlon D. E. Mcnamara G. Sarinopoulos I. C. (2016). Gender differences in justice evaluations: evidence from fMRI. *J. Appl. Psychol.* 101 151–170. 10.1037/apl0000048 26348480

[B20] Eckel C. C. Grossman P. J. (2008). Differences in the economic decisions of men and women: experimental evidence. *Handb. Exp. Econ. Results* 1 509–519. 10.1016/s1574-0722(07)00057-1

[B21] Engel C. (2011). Dictator games: a meta study. *Exp. Econ.* 14 583–610. 10.1007/s10683-011-9283-7

[B22] Ergun S. J. García-Muñoz T. Rivas M. F. (2012). Gender differences in economic experiments. *Rev. Int. Sociol.* 70 99–111. 10.3989/ris.2011.04.19

[B23] Espinosa M. P. Kovářík J. (2015). Prosocial behavior and gender. *Front. Behav. Neurosci.* 9:88. 10.3389/fnbeh.2015.00088 25926783 PMC4396499

[B24] Fehr E. Fischbacher U. (2004). Social norms and human cooperation. *Trends Cogn. Sci.* 8 185–190. 10.1016/j.tics.2004.02.007 15050515

[B25] Feng C. Luo Y. J. Krueger F. (2015). Neural signatures of fairness-related normative decision making in the ultimatum game: a coordinate-based meta-analysis. *Hum. Brain Mapp.* 36 591–602. 10.1002/hbm.22649 25327760 PMC6869807

[B26] Flinkenflogel N. Novin S. Huizinga M. Krabbendam L. (2017). Gender moderates the influence of self-construal priming on fairness considerations. *Front. Psychol.* 8:503. 10.3389/fpsyg.2017.00503 28421019 PMC5376594

[B27] Gabay A. S. Radua J. Kempton M. J. Mehta M. A. (2014). The Ultimatum Game and the brain: a meta-analysis of neuroimaging studies. *Neurosci. Biobehav. Rev.* 47 549–558. 10.1016/j.neubiorev.2014.10.014 25454357

[B28] Gabriel S. Gardner W. L. (1999). Are there “his” and “hers” types of interdependence? The implications of gender differences in collective versus relational interdependence for affect, behavior, and cognition. *J. Pers. Soc. Psychol.* 77 642–655. 10.1037/0022-3514.77.3.642 10510513

[B29] Gelfand M. J. Higgins M. Nishii L. H. Raver J. L. Dominguez A. Murakami F. (2002). Culture and egocentric perceptions of fairness in conflict and negotiation. *J. Appl. Psychol.* 87 833–845. 10.1037//0021-9010.87.5.833 12395808

[B30] Glynn C. J. (1997). Public opinion as a normative opinion process. *Commun. Yearb.* 20 157–183. 10.1080/23808985.1997.11678941

[B31] Gollwitzer M. Bücklein K. (2007). Are “we” more punitive than “me”? Self-construal styles, justice-related attitudes, and punitive judgments. *Soc. Justice Res.* 20 457–478. 10.1007/s11211-007-0051-y

[B32] Gospic K. Mohlin E. Fransson P. Petrovic P. Johannesson M. Ingvar M. (2011). Limbic justice—amygdala involvement in immediate rejection in the ultimatum game. *PLoS Biol.* 9:e1001054. 10.1371/journal.pbio.1001054 21559322 PMC3086869

[B33] Grecucci A. Giorgetta C. Van’t Wout M. Bonini N. Sanfey A. G. (2012). Reappraising the ultimatum: an fMRI study of emotion regulation and decision making. *Cereb. Cortex* 23 399–410. 10.1093/cercor/bhs028 22368088

[B34] Güth W. Kocher M. G. (2014). More than thirty years of ultimatum bargaining experiments: motives, variations, and a survey of the recent literature. *J. Econ. Behav. Organ.* 108 396–409. 10.1016/j.jebo.2014.06.006

[B35] Güth W. Schmittberger R. Schwarze B. (1982). An experimental analysis of ultimatum bargaining. *J. Econ. Behav. Organ.* 3 367–388.

[B36] Han S. Northoff G. (2008). Culture-sensitive neural substrates of human cognition: a transcultural neuroimaging approach. *Nat. Rev. Neurosci.* 9 646–654. 10.1038/nrn2456 18641669

[B37] Han S. Northoff G. Vogeley K. Wexler B. E. Kitayama S. Varnum M. E. (2013). A cultural neuroscience approach to the biosocial nature of the human brain. *Annu. Rev. Psychol.* 64 335–359. 10.1146/annurev-psych-071112-054629 22994921

[B38] Hanel P. H. Vione K. C. (2016). Do student samples provide an accurate estimate of the general public? *PLoS One* 11:e0168354. 10.1371/journal.pone.0168354 28002494 PMC5176168

[B39] Harada T. Li Z. Chiao J. Y. (2010). Differential dorsal and ventral medial prefrontal representations of the implicit self modulated by individualism and collectivism: an fMRI study. *Soc. Neurosci.* 5 257–271. 10.1080/17470910903374895 20178036

[B40] Henrich J. Boyd R. Bowles S. Camerer C. Fehr E. Gintis H. (2001). In search of homo economicus: behavioral experiments in 15 small-scale societies. *Am. Econ. Rev.* 91 73–78. 10.1257/aer.91.2.73

[B41] Henrich J. Heine S. J. Norenzayan A. (2010). Most people are not WEIRD. *Nature* 466:29. 10.1038/466029a 20595995

[B42] Hogg M. A. Reid S. A. (2006). Social identity, self-categorization, and the communication of group norms. *Commun. Theory* 16 7–30. 10.1111/j.1468-2885.2006.00003.x

[B43] Hunt L. T. Kolling N. Soltani A. Woolrich M. W. Rushworth M. F. Behrens T. E. (2012). Mechanisms underlying cortical activity during value-guided choice. *Nat. Neurosci.* 15 470–476. 10.1038/nn.3017 22231429 PMC3378494

[B44] Immordino-Yang M. H. Yang X.-F. Damasio H. (2014). Correlations between social-emotional feelings and anterior insula activity are independent from visceral states but influenced by culture. *Front. Hum. Neurosci.* 8:728. 10.3389/fnhum.2014.00728 25278862 PMC4165215

[B45] Ioannidis J. P. Munafo M. R. Fusar-Poli P. Nosek B. A. David S. P. (2014). Publication and other reporting biases in cognitive sciences: detection, prevalence, and prevention. *Trends Cogn. Sci.* 18 235–241. 10.1016/j.tics.2014.02.010 24656991 PMC4078993

[B46] Jennings R. G. Van Horn J. D. (2012). Publication bias in neuroimaging research: implications for meta-analyses. *Neuroinformatics* 10 67–80. 10.1007/s12021-011-9125-y 21643733 PMC4368431

[B47] Kahneman D. Knetsch J. L. Thaler R. H. (1986). Fairness and the assumptions of economics. *J. Bus.* 59 S285–S300.

[B48] Kashima E. S. Hardie E. A. (2000). The development and validation of the relational, individual, and collective self-aspects (RIC) scale. *Asian. J. Soc. Psychol.* 3 19–48. 10.1111/1467-839x.00053

[B49] Kashima Y. Yamaguchi S. Kim U. Choi S.-C. Gelfand M. J. Yuki M. (1995). Culture, gender, and self: a perspective from individualism-collectivism research. *J. Pers. Soc. Psychol.* 69 925. 10.1037//0022-3514.69.5.925 7473038

[B50] Kirk U. Downar J. Montague P. R. (2011). Interoception drives increased rational decision-making in meditators playing the ultimatum game. *Front. Neurosci.* 5:49. 10.3389/fnins.2011.00049 21559066 PMC3082218

[B51] Kirk U. Gu X. Sharp C. Hula A. Fonagy P. Montague P. R. (2016). Mindfulness training increases cooperative decision making in economic exchanges: evidence from fMRI. *Neuroimage* 138 274–283. 10.1016/j.neuroimage.2016.05.075 27266443 PMC4954868

[B52] Kitayama S. Park J. (2010). Cultural neuroscience of the self: understanding the social grounding of the brain. *Soc. Cogn. Affect. Neurosci.* 5 111–129. 10.1093/scan/nsq052 20592042 PMC2894676

[B53] Klucharev V. Hytönen K. Rijpkema M. Smidts A. Fernández G. (2009). Reinforcement learning signal predicts social conformity. *Neuron* 61 140–151. 10.1016/j.neuron.2008.11.027 19146819

[B54] Kopsida E. Berrebi J. Petrovic P. Ingvar M. (2016). Testosterone administration related differences in brain activation during the Ultimatum Game. *Front. Neurosci.* 10:66. 10.3389/fnins.2016.00066 26973448 PMC4771731

[B55] Kühnen U. Hannover B. Schubert B. (2001). The semantic–procedural interface model of the self: the role of self-knowledge for context-dependent versus context-independent modes of thinking. *J. Pers. Soc. Psychol.* 80 397–409. 10.1037/0022-3514.80.3.397 11300574

[B56] Lemmers-Jansen I. L. J. Krabbendam L. Amodio D. M. Van Doesum N. J. Veltman D. J. Van Lange P. A. M. (2018). Giving others the option of choice: an fMRI study on low-cost cooperation. *Neuropsychologia* 109 1–9. 10.1016/j.neuropsychologia.2017.12.009 29221833

[B57] Loersch C. Payne B. K. (2014). Situated inferences and the what, who, and where of priming. *Soc. Cogn.* 32 137–151. 10.1521/soco.2014.32.supp.137

[B58] Maddux W. W. Brewer M. B. (2005). Gender differences in the relational and collective bases for trust. *Group Process. Int. Relat.* 8 159–171. 10.1177/1368430205051065

[B59] Markus H. R. Kitayama S. (1991). Culture and the self: implications for cognition, emotion, and motivation. *Psychol. Rev.* 98 224–253. 10.1037//0033-295x.98.2.224

[B60] McCabe K. Houser D. Ryan L. Smith V. Trouard T. (2001). A functional imaging study of cooperation in two-person reciprocal exchange. *Proc. Natl. Acad. Sci. U.S.A.* 98 11832–11835. 10.1073/pnas.211415698 11562505 PMC58817

[B61] Ng S. H. Han S. Mao L. Lai J. C. (2010). Dynamic bicultural brains: fMRI study of their flexible neural representation of self and significant others in response to culture primes. *Asian. J. Soc. Psychol.* 13 83–91. 10.1111/j.1467-839x.2010.01303.x

[B62] Oosterbeek H. Sloof R. Van De Kuilen G. (2004). Cultural differences in ultimatum game experiments: evidence from a meta-analysis. *Exp. Econ.* 7 171–188. 10.1023/b:exec.0000026978.14316.74

[B63] Oyserman D. (1993). The lens of personhood: viewing the self and others in a multicultural society. *J. Pers. Soc. Psychol.* 65 993–1009. 10.1037//0022-3514.65.5.993

[B64] Oyserman D. Coon H. M. Kemmelmeier M. (2002). Rethinking individualism and collectivism: evaluation of theoretical assumptions and meta-analyses. *Psychol. Bull.* 128 3–72. 10.1037//0033-2909.128.1.3 11843547

[B65] Oyserman D. Lee S. W. (2008). Does culture influence what and how we think? Effects of priming individualism and collectivism. *Psychol. Bull.* 134 311–342. 10.1037/0033-2909.134.2.311 18298274

[B66] Oyserman D. Novin S. Flinkenflögel N. Krabbendam L. (2014). Integrating culture-as-situated-cognition and neuroscience prediction models. *Cult. Brain* 2 1–26. 10.1007/s40167-014-0016-6

[B67] Phelps E. A. O’connor K. J. Gatenby J. C. Gore J. C. Grillon C. Davis M. (2001). Activation of the left amygdala to a cognitive representation of fear. *Nat. Neurosci.* 4 437–441. 10.1038/86110 11276236

[B68] Preuschoff K. Quartz S. R. Bossaerts P. (2008). Human insula activation reflects risk prediction errors as well as risk. *J. Neurosci.* 28 2745–2752. 10.1523/JNEUROSCI.4286-07.2008 18337404 PMC6670675

[B69] Rilling J. K. King-Casas B. Sanfey A. G. (2008). The neurobiology of social decision-making. *Curr. Opin. Neurobiol.* 18 159–165. 10.1016/j.conb.2008.06.003 18639633

[B70] Rilling J. K. Sanfey A. G. (2011). The neuroscience of social decision-making. *Annu. Rev. Psychol.* 62 23–48. 10.1146/annurev.psych.121208.131647 20822437

[B71] Rilling J. K. Sanfey A. G. Aronson J. A. Nystrom L. E. Cohen J. D. (2004). The neural correlates of theory of mind within interpersonal interactions. *Neuroimage* 22 1694–1703. 10.1016/j.neuroimage.2004.04.015 15275925

[B72] Rimal R. N. Lapinski M. K. (2015). A re-explication of social norms, ten years later. *Commun. Theory* 25 393–409. 10.1186/s12879-018-3571-1 30563503 PMC6299620

[B73] Ruff C. C. Fehr E. (2014). The neurobiology of rewards and values in social decision making. *Nat. Rev. Neurosci.* 15 549–562. 10.1038/nrn3776 24986556

[B74] Sanfey A. G. (2007). Social decision-making: insights from game theory and neuroscience. *Science* 318 598–602. 10.1126/science.1142996 17962552

[B75] Sanfey A. G. Loewenstein G. Mcclure S. M. Cohen J. D. (2006). Neuroeconomics: cross-currents in research on decision-making. *Trends Cogn. Sci.* 10 108–116. 10.1016/j.tics.2006.01.009 16469524

[B76] Sanfey A. G. Rilling J. K. Aronson J. A. Nystrom L. E. Cohen J. D. (2003). The neural basis of economic decision-making in the ultimatum game. *Science* 300 1755–1758. 10.1126/science.1082976 12805551

[B77] Tabibnia G. Satpute A. B. Lieberman M. D. (2008). The sunny side of fairness preference for fairness activates reward circuitry (and disregarding unfairness activates self-control circuitry). *Psychol. Sci.* 19 339–347. 10.1111/j.1467-9280.2008.02091.x 18399886

[B78] Triandis H. C. (1994). *Culture and Social Behavior.* New York, NY: McGraw-Hill.

[B79] Triandis H. C. (1995). “). A theoretical framework for the study of diversity,” in *Diversity in Organizations: New Perspectives for a Changing Workplace*, eds Chemers M. M. Oskamp S. Costanzo M. A. (Thousand Oaks, CA: Sage Publications, Inc), 11–36. 10.4135/9781452243405.n2

[B80] Triandis H. C. (2001). Individualism-collectivism and personality. *J. Pers.* 69 907–924. 10.1111/1467-6494.696169 11767823

[B81] Van Vugt M. De Cremer D. Janssen D. P. (2007). Gender differences in cooperation and competition the male-warrior hypothesis. *Psychol. Sci.* 18 19–23. 10.1111/j.1467-9280.2007.01842.x 17362372

[B82] Wager T. D. Barrett L. F. (2017). From affect to control: functional specialization of the insula in motivation and regulation. *bioRxiv* 102368.

[B83] Wang C. Oyserman D. Liu Q. Li H. Han S. (2013). Accessible cultural mind-set modulates default mode activity: evidence for the culturally situated brain. *Soc. Neurosci.* 8 203–216. 10.1080/17470919.2013.775966 23485156

[B84] Wei Z. Zhao Z. Zheng Y. (2013). Neural mechanisms underlying social conformity in an ultimatum game. *Front. Hum. Neurosci.* 7:896. 10.3389/fnhum.2013.00896 24399954 PMC3872043

[B85] Wiech K. Tracey I. (2009). The influence of negative emotions on pain: behavioral effects and neural mechanisms. *Neuroimage* 47 987–994. 10.1016/j.neuroimage.2009.05.059 19481610

[B86] Ybarra O. Trafimow D. (1998). How priming the private self or collective self affects the relative weights of attitudes and subjective norms. *Personal. Soc. Psychol. Bull.* 24 362–370. 10.1177/0146167298244003

[B87] Zhu Y. Zhang L. Fan J. Han S. (2007). Neural basis of cultural influence on self-representation. *Neuroimage* 34 1310–1316. 10.1016/j.neuroimage.2006.08.047 17134915

[B88] Zinchenko O. Arsalidou M. (2017). Brain responses to social norms: meta-analyses of fMRI studies. *Hum. Brain Mapp.* 39 955–970. 10.1002/hbm.23895 29160930 PMC6866581

